# A nomogram to predict cadmium-induced renal tubular dysfunction

**DOI:** 10.1038/s41598-020-67124-0

**Published:** 2020-06-22

**Authors:** Xinru Wang, Xin Chen, Weiming He, Guoying Zhu, Taiyi Jin, Xiao Chen

**Affiliations:** 1 Department of Radiology, Affiliated Hospital of Nanjing University of Chinese Medicine, Nanjing, 210029 China; 2 Department of Nephrology, Affiliated Hospital of Nanjing University of Chinese Medicine, Nanjing, 210029 China; 3 Institute of Radiation Medicine, Fudan University, 2094 Xietu road, Shanghai, 200032 China; 4 Department of Occupational Medicine, School of Public Health, Fudan University, 150 Dongan road, Shanghai, 200032 China

**Keywords:** Natural hazards, Risk factors

## Abstract

Cadmium-induced renal dysfunction varies between individuals. It would be valuable to figure out those susceptible individuals or predict the risk of cadmium induced renal dysfunction. In the present study, we used a nomogram model to identify high-risk of cadmium-induced renal tubular dysfunction. 342 subjects living in low and moderately cadmium polluted areas were included in this study. The daily cadmium intake from food (FCd) was estimated using food survey. The cadmium in blood (BCd) and urine (UCd) were detected by using flame atomic absorption spectrometry. Urinary β_2_Microglobulin (UBMG) was chosen as indicator of renal dysfunction. Logistic regression was used to select the independent risk factors for renal dysfunction. Bootstrap self-sampling and calibration curves were performed to quantify our modeling strategy. Age, sex, BCd and TCd were used to construct the nomogam in total population; age, BCd and TCd were adopted in women; age and BCd were used in men. The internal validation showed the C-index was 0.76 (95% 47 confidence interval (CI): 0.71–0.82) in total population, 0.74 (95% CI: 0.69–0.79) in men and 0.78 (95% CI: 0.72–0.84) in women. The area under the curve of the nomogram was 0.77 (95% CI: 0.71–0.83) in total population, 0.82(95% CI: 0.74–0.90) in women and 0.74(95% CI: 0.66–0.82) in men. Nomogram may be a rapid and simple risk assessment tool for predicting high-risk of renal tubular dysfunction in subjects exposed cadmium.

## Introduction

Cadmium, a persistent environmental heavy metal, is one type toxicant to human. Cadmium contamination in farmland and food, such as rice and leaf vegetables, is one of major concerns in China^1^. A recent report showed that Cd contamination was found in 7% of the total soil survey sites^2^. Cadmium can accumulate in the internal organs and cause damage to many systems. Kidney is the target organ for cadmium toxicity. Moreover, the biological half-life of cadmium in kidney is long, 10–30 years^3^.

The cadmium-induced renal dysfunction has been widely investigated. The cadmium-induced renal tubular function can be evaluated by biomarker, such as Urinary β_2_Microglobulin (UBMG), urinary N-acetyl-β-D-glucosaminidase (UNAG) and Kim^1^. However, cadmium-induced renal tubular dysfunctions are usually asymptomatic. In addition, renal tubular biomarkers are not usually determined in physical examination. Moreover, the studies in China and Japan both indicated that renal tubular function remained normal in some subjects with high level of urinary cadmium (UCd). Jin *et al*.^4,5^ showed that almost 50% of subjects with high UCd [> 20 μg/g creatinine (Cr)] showed normal renal tubular dysfunction, as shown normal UNAG and UBMG. Similar results were observed in several other Chinese studies^6,7^. Several studies in Japan also showed that renal tubular dysfunction was only observed in 20–32% of subjects living in cadmium polluted area^8^ or 22–70% of female subjects with UCd of 5–30 μg/g cr^9^. Those data indicated that cadmium-induced renal dysfunction varies between individuals^10^. Many factors may affect the cadmium toxicity to kidney, such as exposure levels, age and diabetes^11,12^. It would be interesting and valuable to establish a model to predict the renal tubular dysfunction in those population living in cadmium polluted area. However, to our best knowledge, few study have mentioned those models.

Many models has been used in clinical studies to predict cancer metastasis, efficiency of chemotherapy, cancer recurrences and prognosis^13,14^. In recent years, nomogram is one of the widely accepted models. It is graphical calculating model which can accurately calculate the individual risk events by synthetically considering all independent risk factors^15,16^. However, this model rarely used in field of environmental health. In addition, no study have used this model to predict cadmium-related health risk. In the present study, we aimed to show the value of nomogram in predicting cadmium-induced renal dysfunction in a Chinese population.

## Results

### The characteristics of subjects

The characteristics of subjects are list in Table 1. There were 173 women and 169 men. The mean of age was 45.9 ± 11.3 (46.1 for women and 45.6 for men) years old. The median levels of TCd, FCd, BCd and UCd were 141.0 μg/d, 126.4 μg/d, 7.03 μg/L and 8.30 μg/g cr. The median UBMG was 0.166 mg/g cr and renal tubular dysfunction (UBMG > 0.5 mg/g cr) was observed in 23.1% subjects. The BCd and UCd level in women was lower and higher than these in men, respectively, but no significant differences were found.

**Table 1:** Characteristics of Subject.

Data	Total (n = 342)	Women (n = 173)	Men (n = 169)
Age (y)	45.9 ± 11.3	46.1 ± 11.1	45.6 ± 11.4
FCd (μg/d)	126.4 ± 49.5	127.9 ± 49.9	124.9 ± 49.1
TCd (μg/d)	141.0 ± 64.0	127.8 ± 49.9	154.5 ± 73.5
BCd (μg/d)	7.03(0.19–75.9)	6.0(0.19–75.9)	8.0(0.37–61.2)
UCd (μg/g cr)	8.30(0.02–87.7)	9.1(0.08–87.7)	7.2(0.02–63.9)
BMI (kg/m2)	23.2 ± 4.6	22.4 ± 4.3	24.1 ± 5.2
UBMG (mg/g cr)	0.17(0.01–9.5)	0.14(0.02–8.4)	0.24(0.01–9.53)
UBMG > 0.8	79(23.1%)	35(20.0%)	42(25.0%)

BCd: cadmium in blood; BMI: body mass index; Cr: creatinine; FCd: cadmium in food; TCd: total cadmium intake;UCd: urinary cadmium; UBMG: urinary β2Microglobulin.

### The associations between risk factors and tubular dysfunction

The associations between risk factors and tubular dysfunction were analyzed using 168 logistic regression analysis (Table 2). Age, sex, BCd and FCd were independent risk factors of renal tubular dysfunction in all population. The odds ratio (OR) was 1.04 (95% CI: 170 1.01–1.07), 1.81(95% CI 1.07–3.06), 1.04 (95% CI: 1.01–1.07), and 1.01(95% CI: 171 1.01–1.02), respectively. Similar results were found in women and men.

**Table 2:** Odds ratios (ORs) and 95% confidence intervals (CIs) of renal dysfunction and risk factors.

	Tubular dysfunction		
	Total	Women	Men
Age (y)	1.04(1.01–1.07)	1.04(1.00–1.09)	1.03(1.00–1.06)
BCd (μg/L)	1.04(1.01–1.07)	1.07(1.02–1.12)	1.03(1.00–1.08)
TCd(μg/d)	1.01(1.00–1.02)	1,02(1.01–1.04)	1.01(0.99–1.01)
Gender(Male vs Female)	1.81(1.07–3.06)		
Smoking	1.06(0.94–1.32)		1.82(0.0.68–4.89)
Drinking	0.89(0.79–1.13)		0.84(0.72–1.10)

BCd: cadmium in blood; FCd: cadmium in food; TCd: total cadmium intake.

#### Nomogram model

**Figure 1:**
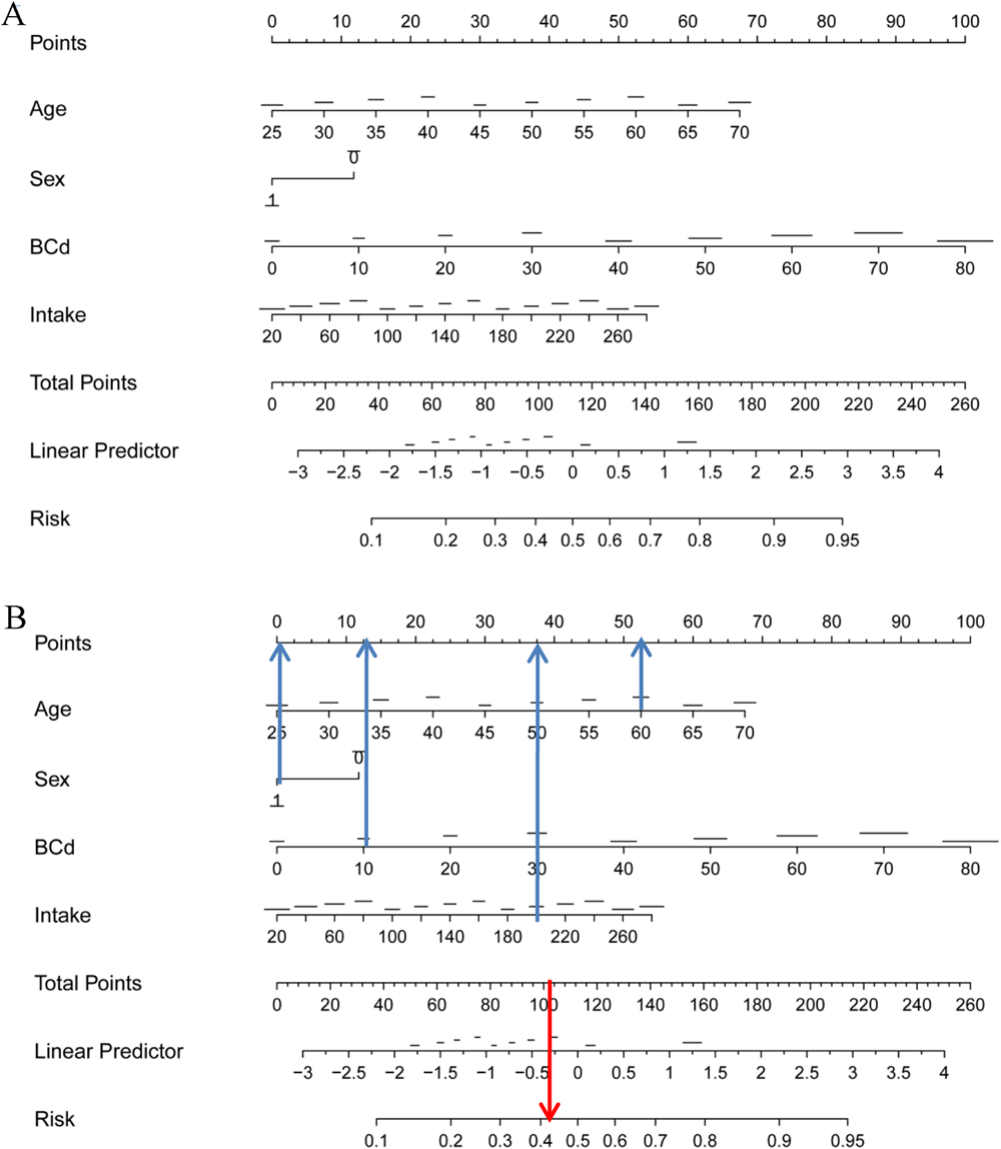
The nomogram for subjects with different levels of cadmium exposure (**A**) and an example on how to use the nomogram (**B**). Each variables is assigned a score on the points scale. The sum of each score was obtained as total point. The total points correspond to the estimated probability for renal tubular dysfunction. A 60-year-old woman with BCd of 10 μg/L and FCd of 200 μg/d would have 52.5 points for his age, 12 points for the BCd, 37.5 points for cadmium intake, and 0 point for gender. The total point was 102. The total points correspond to the risk of 0.42 for renal tubular dysfunction.

**Figure 2:**
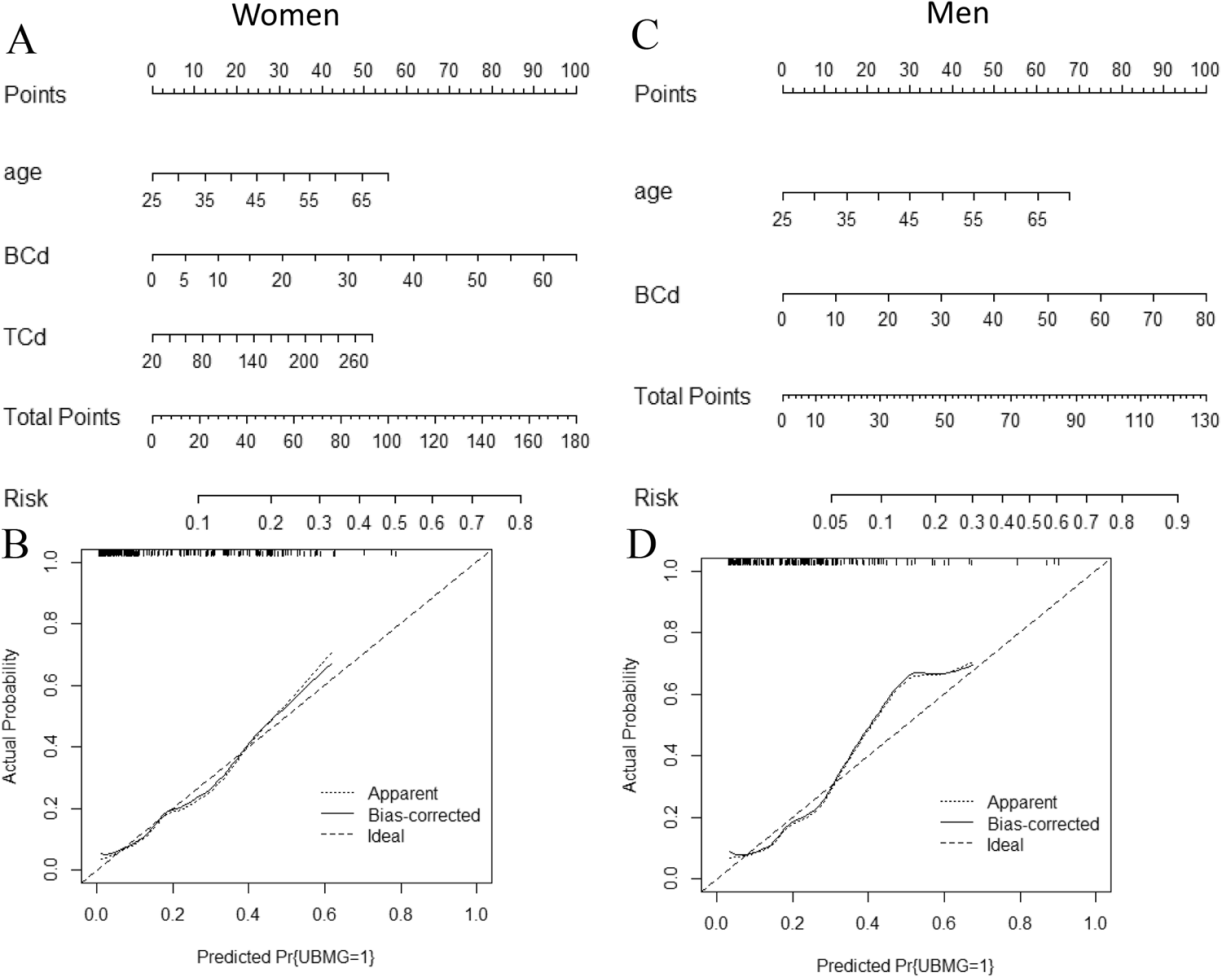
The nomogram and calibration plots for female (**A**) and male (**B**) subjects with different levels of cadmium exposure. BCd: cadmium in blood; FCd: cadmium in food; TCd: total cadmium intake.

**Figure 3:**
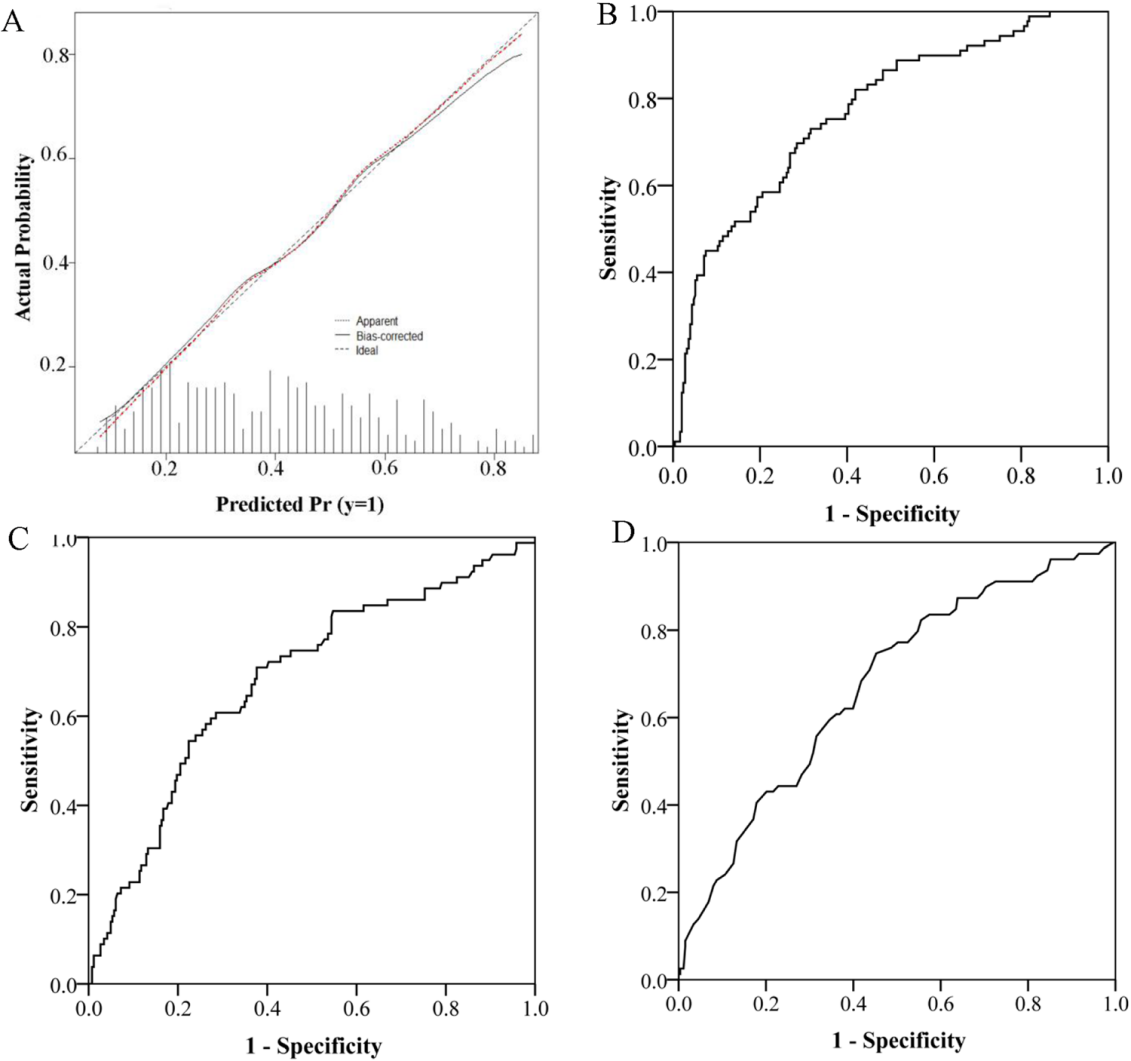
The calibration plots of the nomogram (**A**) using bootstrapping with 1,000 resamples and Receiver operating characteristics (ROC) curves of nomogram model (**B**), BCd (**C**) and FCd (**D**). BCd: cadmium in blood; FCd: cadmium in food.

Age, BCd and UCd were finally recruited into the nomogram model to predict the risk of renal tubular dysfunction in total population (Fig. 1) and women(Fig. 2). For men, age and BCd were finally recruited into the nomogram model (Fig. 2). The nomogram demonstrated that BCd contributed the most to risk of renal tubular dysfunction. Every variable was assigned a score on the points scale. A total score was obtained from the sum of each variables. The risk was shown in a probality scale. The calibration plots of the nomogram are shown in Fig. 2 and Fig. 3A using bootstrapping with 1,000 resamples. The nomogram was with a C-index of 0.76 [95% confidence interval (CI): 0.71–0.82] in total population, 0.74 (95% CI: 0.69–0.79) in women and 0.78 (95% CI: 0.72–0.84).

We showed an example of how to use the nomogram (Fig. 1B). A 60-year-old woman with BCd of 10 μg/L and TCd of 200 μg/d would have 52.5 points for his age, 37.5 points for the BCd, 12 points for cadmium intake and 0 points for gender. The total point was 102. The total points correspond to the risk of 0.42 for renal tubular dysfunction. For a 60-year-old woman with low or no cadmium exposure, the risk was lower than 0.2.

#### Predictive performance

**Figure 4:**
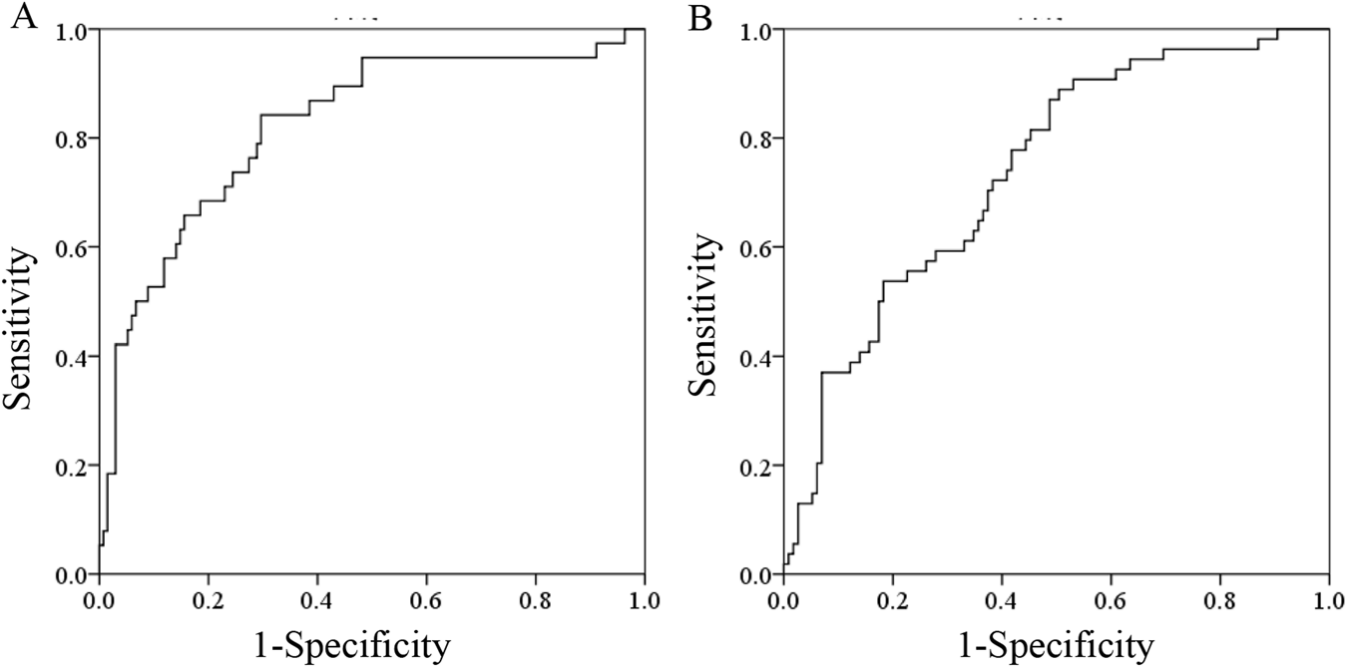
Receiver operating characteristics (ROC) curves of nomogram model in women and men in predicting risk of renal dysfunction. The area under the curve of the nomogram was 0.82(95% CI: 0.74–0.90) in women and 0.74(95% CI: 0.66–0.82) in men.

Receiver operating characteristics (ROC) curves are shown in Figs. 3B–D and 4. The area under the curve (AUC) values of nomogram model, BCd and FCd were 0.77 (95% CI: 0.71–0.83), 0.68 (95% CI: 0.61–0.75) and 0.67 (95% CI: 0.60–0.74) in total population, respectively. The area under curve (AUC) of the nomogram was 0.82 (95% CI: 0.74–0.90) in women and 0.74 (95% CI: 0.66–0.82) in men.

## Discussion

Renal tubular dysfunction is one of the harmful effects for cadmium. However, renal tubular dysfunction varies between individuals. In this study, we developed a nomogram model to predict the risk of cadmium-induced renal dysfunction with based on the independent risk factors. C-index and calibration curves all showed that the nomogram had acceptable predictive performance. The nomogram may be a valuable tool in predicting health risk of cadmium toxicity.

Many studies have reported the adverse effects of cadmium on renal function^4,11^. The prevalence of renal tubular dysfunction increased with the increased BCd or UCd. Interestingly, we observed that some subjects with low to moderate level of cadmium exposure did not occur renal dysfunction. Many factors, including environmental factors and genetic factors, may modify susceptibility to kidney injury^17^. Horiguchi *et al*.^12^ reported that aging process may be one of determinant for cadmium-related renal effects. In the present study, we also showed that age was an independent risk factor for renal dysfunction in subjects exposed to cadmium. Age is equal to the exposure time in our population because the cadmium pollution existed hundreds years in this areas. Smoking may be another factors for cadmium related renal dysfunction^18^. However, no such association was observed in our study. One explanation was that the cadmium exposure from food was much higher than tobacco in our population.

Logistic model can be used to identify risk factors and may be also used to predict the health risk. However, this model is not intuitive. Recent studies showed that a nomogram can visually interpret the logistic regression model^19^. It is shown as a chart or graph of scaled variables^15,19^. Those variables can be obtained based on the regression models. Nomograms have the advantage of graphical interpretation of numeric statistics. It can provides quick and simple personalized prediction of risk or prognosis. Therefore, more and more clinical studies used this model to predict survival or prognosis of diseases^20–22^. However, this model has not been widely used in the field of environmental health. Only few studies used this model in field of toxicology^23^. Our study is one exploration. Three components, age, BCd and TCd, were included in our nomogram based on the logistic regression model. ROC analysis showed that the predictive value of nomogram model was better than BCd or FCd. Physicians may use this nomogram to provide a numerical estimation of risk of cadmium-induced renal tubular dysfunction.

There are several limitations in our study. First, our study was conducted in a single cadmium polluted area. The risk factors may vary in different populations. An external validation was required to confirm our finding. Second, our population was environmentally exposed to cadmium. Additional studies are needed to test whether our model is fit for those subjects with occupational cadmium exposure. Third, the population may co-expose to cadmium and zinc or lead. However, those cofounders were not considered in the present study. Finally, we did not observed the association between cadmium exposure and chronic kidney diseases (CKD) because tubular dysfunction was more common than CKD in populations that had cadmium exposure^24^.

In conclusion, our data showed that age, BCd and FCd are the independent risk factors of cadmium-related renal tubular dysfunction. Nomogram may be a valuable numerical tool for risk prediction of renal tubular dysfunction in subjects exposed to cadmium. In addition, nomogram may be useful in personalized health risk assessment.

## Methods

### Study area and population

The study area and population have been reported in our previous studies^6,25,26^. Briefly, two areas located in southern China were included in this study: a low and a moderately cadmium polluted area (Dayu County). Dayu county is one of the important tungsten-ti mining area in China. Large quantities of waste water was directly discharged into the surrounding river which was the source of irrigation for the rice fields. The cadmium concentration in unpolished rice and vegetables were 0.59 mg/kg and 0.06–0.66 mg/kg^25^, respectively, which were both higher than National standard (0.2 mg/kg and 0.05–0.2 mg/kg). An area 20 km away from Dayu that had similar living conditions, social and economic conditions and lifestyle was selected as the control area. The cadmium concentration in polished rice were below the national standard of China (0.08 mg/kg in rice) and 0.02–0.14 mg/kg in vegetables).

During the epidemiological survey, only those subjects that had lived in these areas for at least 25 years were included. Those subjects with diabetes and cardiovascular diseases were excluded. Total of 342 local residents were included in the final analysis. We collected the demographic information, cigarette smoking habits and living conditions, and medical history via a questionnaire. The study was conducted ethically in accordance with the World Medical Association Declaration of Helsinki. The study was approved by Institutional Review Board of Fudan University (Shanghai, China). Informed consent was obtained from each participant.

### Sample collection and Exposure analysis

Urine and blood samples were collected from each subjects. The detailed information for sample collection were reported in several previous reports^6,25,26^. Briefly, blood and urine samples were collected and frozen at −20 °C until analysis in local laboratory. UCd and cadmium in blood (BCd) were digested using concentrated nitric acid. The cadmium concentration were determined by using flame atomic absorption spectrometry (AAS)^25^. The coefficient of variation was all below 5% and the recovery rate was 92–108%.

The cadmium intake was estimated as described in the previous studies^6,25,26^. In brief, the information of weekly food consumption, including the type and amount of main foods, was obtained via the questionnaire. 60 households were selected in the two areas. Each types of food obtained from the same area were pooled together for cadmium analysis. Local tobacco was also collected for cadmium determination. All samples were ashed and dissolved in nitric acid. The cadmium was determined using flame AAS or graphite furnace AAS (Perkin Elme, Model PE-3030). The total daily cadmium intake (TCd) was estimated using the following equations: cadmium intake = Σ (cadmium in food (FCd) × food consumption × weighting factor × 0.05) + cadmium in tobacco × tobacco consumption × 0.03. The weighting factor for daily intake to be 1 for ages 21–60 years; 0.41 for ages <10 years, 0.89 for ages 11–20 years, and 0.82 for ages: ≥ 60 years (Cai *et al*., 1998). The fractional uptake of cadmium from food and smoking was set as 0.05 and 0.03, respectively. The differences in cadmium intake between males and females were not considered because previous reported showed the differences was small (Cai *et al*., 1998).

### Renal markers determination

Urinary β_2_Microglobulin (UBMG) was chosen as renal tubular indicator. The detailed information for the measurement was described in the previous reports^25^. Briefly, the UBMG was determined using radioimmunoassay (RIA) kits obtained from Chinese Academy of Science. The urinary creatinine (UCr) were also determined using Jaffe reaction method. UCd and UBMG were adjusted with UCr and expressed as milligram/g cr or microgram/g cr. The cut-off value of 0.5 mg/g cr was selected. UBMG levels above the cut-off values were regarded as “renal tubular dysfunction.”

### Statistical analysis

Data was managed and analyzed using commercial statistical software (SPSS Version 16.0 or R version 3.6.4). Data were shown as mean ± standard variation (normal distribution) or median (range) (abnormal distribution). The two-tailed t tests and Mann-Whitney U-test were used to compare the difference. Logistic regression were adopted to show the association between risk factors [including age, gender, UCd, BCd, TCd, body mass index (BMI), alcohol drinking and smoking] and renal tubular dysfunction. Nomogram model was established using R software. Harrell’s concordance index (C-index) and Bootstrap self-sampling were used to evaluate the predictive performance of nomogram model. Receiver operating characteristics (ROC) curves was used to show the performance of nomogram, FCd, and BCd in predicting renal dysfunction. P < 0.05 was considered statistically significant.

## Data Availability

All data generated or analyzed during this study are included in this published article (and its Supplementary Information files).
